# Two-Year Outcome From Combining Cryoballoon Ablation and Left Atrial Appendage Closure: CLACBAC Study

**DOI:** 10.3389/fcvm.2020.610537

**Published:** 2021-01-11

**Authors:** Zhongyuan Ren, Jingying Zhang, Songyun Wang, Peng Jia, Xiang Li, Jun Zhang, Rong Guo, Hailing Li, Shuang Li, Haotian Yang, Yixing Zheng, Weilun Meng, Yawei Xu, Dongdong Zhao

**Affiliations:** ^1^Department of Cardiology, Shanghai Tenth People's Hospital, Tongji University School of Medicine, Shanghai, China; ^2^Soochow University Medical College, Suzhou, China; ^3^Department of Cardiology, Renmin Hospital of Wuhan University, Wuhan University School of Medicine, Wuhan, China

**Keywords:** combined procedure, cryoballoon ablation, left atrial appendage (LAA) closure, prognose, atrial fbrillation

## Abstract

**Objective:** Catheter ablation combined with left atrial appendage closure (LAAC) has emerged as a promising strategy for atrial fibrillation (AF) patients at high risk for stroke or with contraindications for oral anticoagulants (OACs). But the evidence for the long-term safety and efficacy of a combined procedure using cryoballoon ablation (CBA) with LAAC is still insufficient.

**Methods:** From October 2015 to December 2017, a total of 76 consecutive non-valvular, drug-refractory AF patients who underwent a combined procedure of CBA and LAAC are included. Peri- and post-procedural safety and efficacy were evaluated through scheduled follow-ups and transesophageal echocardiography (TEE).

**Results:** A total of 74 patients (97.4%) underwent the combined procedure and achieved instant pulmonary vein isolation and satisfactory LAAC. With a mean follow-up time of 23.7 ± 11.0 months, the recurrence of atrial arrhythmia was recorded in 35 patients (48.0%). In addition, a survival analysis shows a non-significant higher recurrence in persistent AF (*p* = 0.48). The overall OAC withdrawal rate was 97.2%, and one patient (1.4%) had a lethal hemorrhagic stroke while on single antiplatelet therapy. For safety concerns, the overall mortality was 2.7%, which resulted from one case of myocardial infarction on OAC and one hemorrhagic stroke, as mentioned. No other major hemorrhagic events occurred. Among the 72 patients (94.7%) who underwent TEE, one patient (1.4%) had device-related thrombosis and one patient (1.4%) had prominent residual flow (over 3 mm). Both were prescribed long-term OACs without severe complications occurring.

**Conclusions:** Combining CBA with LAAC in a single procedure achieved considerable long-term safety and efficacy, providing a promising strategy for AF management.

## Introduction

Atrial fibrillation (AF) is a chronic degenerative cardiac disease with a prevalence over 10 million in China (1). Furthermore, AF is a serious issue in the Chinese population, where studies have reported insufficient anticoagulation and rhythm control due to low compliance (2). AF poses an enormous health burden and necessitates effective management.

Since pulmonary vein isolation (PVI) was established as the benchmark for AF control (3), AF can be effectively treated by catheter ablation (CA) with minimal lesions (4, 5). Among various novel energy sources, cryoballoon ablation (CBA) provides a promising non-inferior alternative to conventional radiofrequency (RF) ablation (5) with less exposure time and a smoother learning curve (6).

As left atrial appendage (LAA) harbors 90% of thrombus non-valvular AF patients (7), LAA closure (LAAC) was invented to prevent stroke, and studies have validated its safety and efficacy compared to oral anticoagulants (OACs) (8). A European consensus on LAAC proclaimed in 2019 that LAAC could be a choice for stroke prophylaxis if non-compliance impeded anticoagulation treatment (9), and it recognized LAAC as a feasible approach for AF management.

To achieve integrated AF control and stroke prevention, Swaans et al. (10) reported the first attempt at combining CA with LAAC in a single procedure that was successful in all 30 symptomatic drug-refractory AF patients who were at a high risk of stroke or had contraindications to OACs. Follow-up evidence proved the safety and efficacy of both RF ablation (11–13) and the CBA (14, 15) combined procedure. The current evidence, however, is less supportive due to limited sample sizes and study design, as well as the lack of standardized indications and varying postprocedural antithrombotic therapies.

Hence, from this registered study, we seek to provide evidence for the safety and efficacy of combining CBA and LAAC through the long-term follow-up of clinical events and scheduled transesophageal echocardiography (TEE) evaluations.

## Materials and Methods

### Studied Population

Based on the registered trial, Combining Left Atrial Appendage Closure With Cryoballoon Ablation in the Chinese Population (CLACBAC, registration number NCT04185142), from October 2015 to December 2017, we investigated patients with documented drug-refractory, non-valvular AF who underwent the combined procedure of CBA and LAAC. AF is defined as absolutely irregular RR intervals and no discernible, distinct P waves confirmed by a 12-lead electrocardiograph (ECG) or Holter. Paroxysmal AF (PAF) is defined as an AF converted to a sinus rhythm spontaneously or by intervention [*via* cardioversion or antiarrhythmic drugs (AADs)] within 7 days, while the persistent type (persAF) is defined as that which lasts longer than 7 days, including episodes that are terminated by intervention after 7 days or more (4). Patients are considered eligible for the combined procedure if they meet at least one of the following criteria:

- CHA_2_DS_2_-VASc score ≥2 and/or HAS-BLED score ≥3
- contraindications to long-term OACs (active major hemorrhagic diseases, inherited hemorrhagic disorders, or severe side effects under OACs)
- refusal of OACs according to personal willingness despite comprehensive explanation.

**Figure 1:**
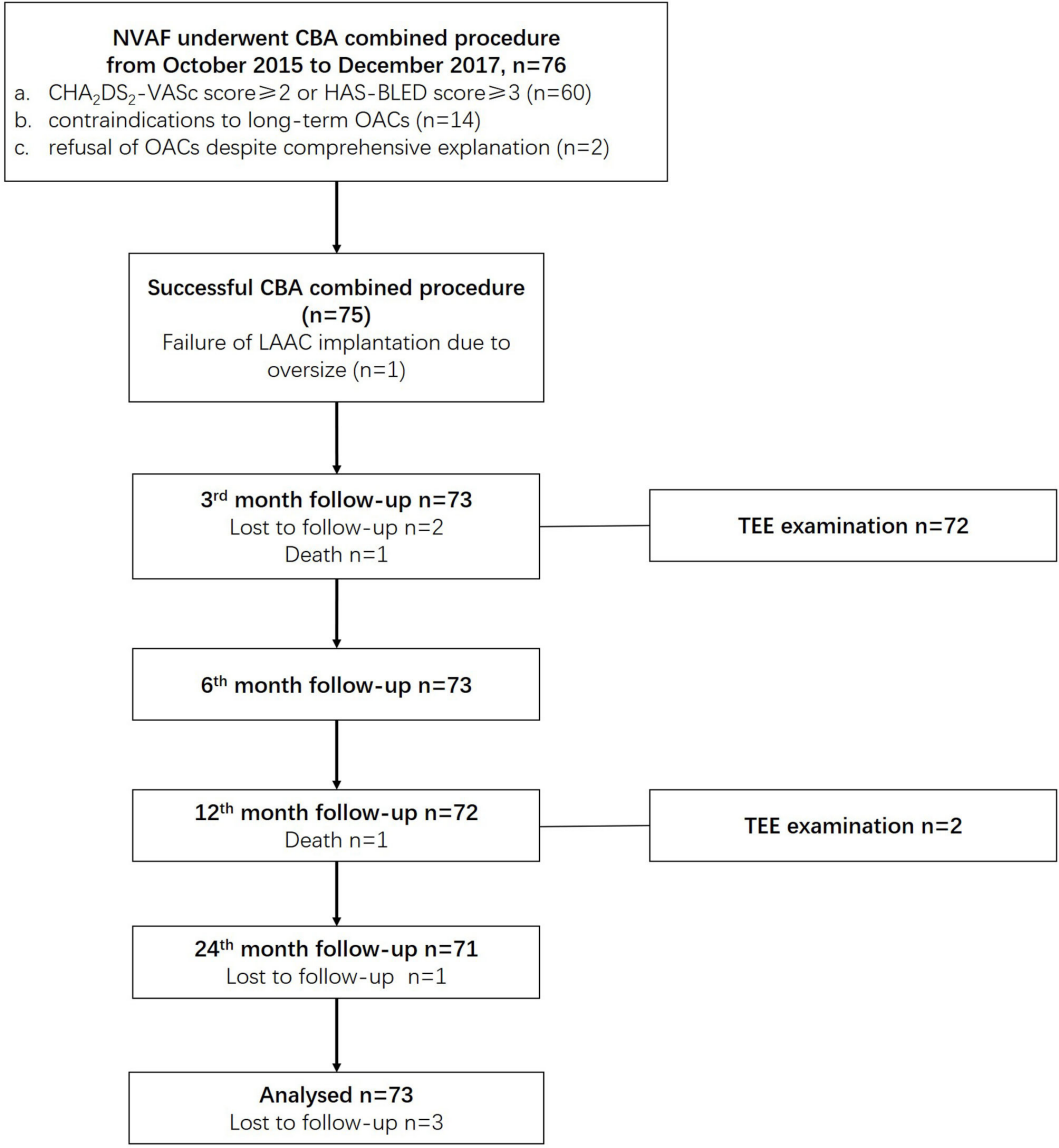
Overall study schemes. CBA, cryoballoon ablation; LAAC, left atrial appendage closure; NVAF, non-valvular atrial fibrillation; OACs, oral anticoagulants.

For every patient, a consent form was signed before the procedure after he or she was fully informed of the procedural risks and related complications. This study complies with the Helsinki declaration and was approved by the ethics committee of Shanghai Tenth People's Hospital. The combined procedure was performed by proficient electrophysiologists in our center who perform over 100 cases of CBA and 50 cases of LAAC per year. The overall study scheme is presented in Figure 1.

### Procedure Details

#### Cryoballoon Ablation

**Figure 2:**
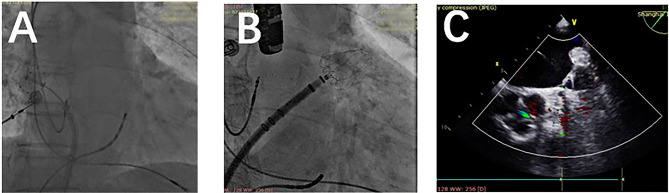
Fluoroscopy and transesophageal echocardiography (TEE) during the combined cryoballoon ablation (CBA) procedure. **(A)** During CBA, an inflated cryoballoon during the ablating of the left superior pulmonary vein. **(B)** Deploying a WATCHMAN device during left atrial appendage closure. **(C)** TEE confirmation of complete sealing stable positioning.

The CBA procedure was performed under local anesthesia in the groin region following transvenous puncture *via* the femoral vein (16). Briefly, first- and second-generation CB (Arctic Front, Medtronic, MN, USA) was utilized for the first 38 and the remaining 38 patients, respectively. Under digital subtraction angiography (DSA) and through femoral access, a Swartz Sheath was advanced into the right atrium, and a transseptal puncture was performed using a puncture needle. Once a pulmonary vein (PV) was confirmed, a CB was inflated and advanced to the ostium of the PV through a steerable sheath (FlexCath, Medtronic, MN, USA), which was followed by angiography to ensure complete occlusion. An Achieve catheter (Achieve; Medtronic, Minneapolis, MN) was cannulated distally to detect electric activity and monitor PVI. A time-to-isolation (TTI) freezing strategy was adopted as we previously described (16) (Figure 2A). The freezing time was adjusted to 150–180 and 180 s when TTI ≤30 s and when TTI was between 30 and 60 s, respectively. If TTI was > 60 s, a 180-s freeze with a bonus 120-s freeze was applied. If TTI was not recorded, a 180-s freeze was applied, or a 180-s freeze with a bonus 120-s freeze was adopted if temperatures fell below−40°C before 60 s after freezing. The phrenic nerve was protected using phrenic pacing (8–10 V at a pace interval of 2,000 ms) during the freezing of the right superior PV (RSPV) and the right inferior PV (RIPV). Under fluoroscopy, phrenic nerve palsy was monitored through diaphragm movement.

For esophageal protection and to avoid serious complications (e.g., atrial esophageal fistula), freezing was halted once patients developed severe nausea with vomiting or complained of intolerable chest distress. An activated clotting time (ACT) of over 300 s was monitored and maintained during the procedure.

#### Left Atrial Appendage Closure

**Figure 3:**
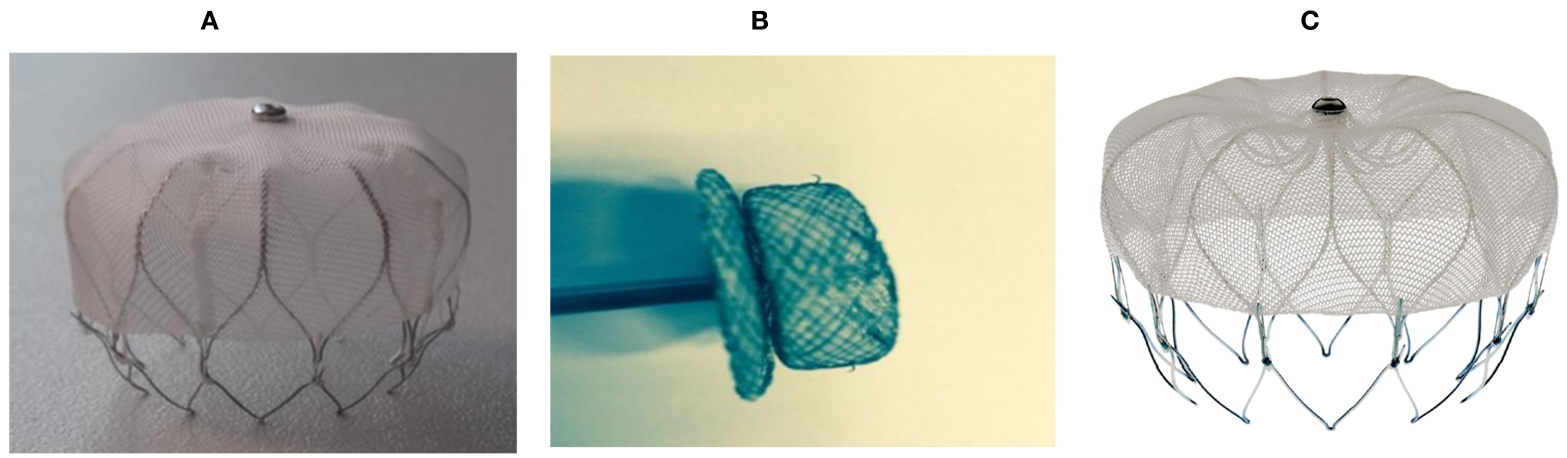
Left atrial appendage closure devices. **(A)** The Lefort device (Shape Memory Alloy Co., Shanghai, China), **(B)** the Lacbes device (Shanghai Push Medical Device Technology Co., Ltd., Shanghai, China), and **(C)** the WATCHMAN device (WATCHMAN, Boston Scientific, MA, USA).

The LAAC device was implanted instantly after CBA. Three types of LAAC devices were used: the Lefort (Shape Memory Alloy Co., Shanghai, China), Lacbes (Shanghai Push Medical Device Technology Co., Ltd., Shanghai, China), and WATCHMAN devices (WATCHMAN, Boston Scientific, MA, USA) (Figure 3). After simple randomization, 39, 17, and 20 patients were given Lefort, Lacbes, and WATCHMAN device implants, respectively. Sharing the same venous access and according to the measured size, depth, and shape of LAA under DSA and TEE, an oversized device (110–120% of the LAA) was chosen to ensure stable positioning and proper compression. A 14-F sheath was advanced into the LAA. Subsequently, the selected device was carefully delivered in the LAA through the access system and deployed at the ostium of the LAA by retracting the access sheath. Before release, the PASS principle was applied and confirmed by the device's position; stable anchoring was confirmed by the tug test, an appropriate compression ratio (10%−25% of the original size), and complete sealing (residual flow ≤ 3 mm). Once released, LA angiography and TEE were applied to reconfirm device implantation and evaluate complications such as pericardial effusion. LAAC procedure fluoroscopy and TEE are presented in Figures 2B,C.

### Follow-Up

Primary endpoints were composed of the recurrence of atrial arrhythmia (AA) and the incidence of stroke. AA recurrence was defined as recorded AF, atrial flutter (AFL), or atrial tachycardia (AT) lasting longer than 30 s after the third month since the procedure. The first 3 months were defined as a blank period when AA episodes were not considered to be a recurrence. The continued use of AADs was not considered as recurrence in this study. Stroke (ischemic or hemorrhagic) was confirmed by either CT or MRI diagnosis.

Secondary endpoints were composed of death by all causes, major hemorrhagic events, systemic embolism, redo-ablation, rehospitalization, withdrawal of OACs, pericardial effusion, atrioesophageal fistula, and device-related events [positioning, residual flow, and device-related thrombosis (DRT)].

All patients were required to have outpatient follow-ups in the first, third, sixth, and 12th months, as well as every year after the procedure. Additionally, transtelephonic follow-ups were applied every 3 months to observe clinical outcomes. During outpatient follow-ups, comprehensive medical histories were acquired and physical examinations were conducted; a Holter was used to detect AA recurrence, and imaging examinations were conducted if necessary. TEE was scheduled for the 3rd month and the 12th month, if necessary, to evaluate device positioning, residual flow, DRT, and other complications.

Antithrombotic therapy was recommended as follows: (1) 3 months of OACs (warfarin, dabigatran, or rivaroxaban), (2) double antiplatelet therapy (DAPT) for another 3 months according to the TEE examination in the third month, and (3) lifelong single antiplatelet therapy (SAPT).

### Statistical Analysis

Continuous variables were described as mean ± standard deviation (SD) if they conformed to a normal distribution, while those without a normal distribution were presented as median with interquartile ranges. Categorical variables were described as percentages (%). For survival analysis, a Kaplan–Meier estimate analyzed the freedom from AA. *P*-values between subgroups were generated from the log-rank test. Two-sided *P* < 0.05 were considered significant for all analyses. The SAS 9.4 software (SAS Institute Inc., Cary, NC, USA) was adopted to conduct all analyses.

## Results

Among the 76 consecutive patients, 60 underwent the combined CBA procedure with high stroke or bleeding risk; 14 had contraindications to long-term OACs, and two refused OAC therapy. Nearly one third of the cohort was composed of persAF with high stroke (a CHA_2_DS_2_-VASc score of 3.4 ± 1.9) and bleeding risks (a HAS-BLED score of 2.3 ± 1.1). Transthoracic echocardiography (TTE) revealed a relatively large left atrium (44.9 ± 5.5 mm). The general condition was significant, with good heart function and mild renal dysfunction. Of note, anticoagulation was far from sufficient, with less than half of the patients taking OACs. Detailed baseline characteristics are listed in Table 1.

**Table 1:** Detailed baseline information.

**Parameters**	**Combined procedure (*N* = 76)**
Age, years	67.0 ± 7.5
Gender (female), n (%)	29 (38.7)
BMI, kg/m^2^	26.1 ± 3.3
AF type (persAF), n (%)	25 (32.9)
HAS-BLED score	2.3 ± 1.1
<3, n (%)	45 (59.2)
≥3, n (%)	32 (42.1)
CHA_2_DS_2_-VASc score	3.4 ± 1.9
<2, n (%)	16 (21.1)
≥2, n (%)	60 (78.9)
proBNP, pg/ml	496.4 (265.9, 859.4)
eGFR, ml/(min * 1.73 m^2^)	78.5 ± 16.1
Left atrial diameter, mm	44.9 ± 5.5
LVEF, %	60.2 ± 7.4
**Medical history**	
Hypertension, n (%)	49 (64.5)
Diabetes mellitus, n (%)	17 (22.4)
Heart failure, n (%)	8 (10.5)
Coronary heart disease, n (%)	15 (19.7)
Previous PCI, n (%)	6 (7.9)
Stroke history, n (%)	34 (44.7)
Hemorrhage history, n (%)	4 (5.3)
**Medications**	
Oral anticoagulants, n (%)	37 (48.7)
Warfarin, n (%)	20 (26.3)
New oral anticoagulants, n (%)	17 (22.7)
Antiplatelet agents, n (%)	16 (21.1)

*Continuous variables are presented as mean ± SD or as median with an interquartile range; categorical variables are reported as a percentage (%). BMI, body mass index; eGFR, estimated glomerular filtration rate (calculated via CKD-EPI formula); LVEF, left ventricular ejection fraction; PCI, percutaneous coronary intervention; persAF, persistent atrial fibrillation; proBNP, pro brain natriuretic peptide*.

### Procedure Details

On the one hand, the overall success rate of the combined CBA procedure was 97.4%, resulting from one patient who failed to achieve instant isolation of RIPV and another patient whose LAAC device implantation failed due to oversized LAA. On the other hand, the procedure was performed safely for all patients, with only five of them experiencing a vagal reflex during CBA; 15 had minimal residual flow (≤ 3 mm) detected by TEE after LAAC. No other complications like cardiac tamponade or phrenic nerve palsy were observed throughout the entire procedure. Detailed parameters are listed in Table 2.

**Table 2:** The periprocedural parameters of the combined procedure.

**Procedural parameters**	**Combined procedure (*N* = 76)**
**Cryoballoon ablation**	
Second-generation cryoballoon, n (%)	38 (50.0)
Instant pulmonary vein isolation, n (%)	75 (98.7)
**Lowest temperature**, **°C**	
LSPV	−50.6 ± 4.9
LIPV	−45.7 ± 5.2
RSPV	−51.6 ± 7.1
RIPV	−45.0 ± 4.8
**Freezing duration, s**	
LSPV	249.5 ± 155.3
LIPV	248.4 ± 108.3
RSPV	183.1 ± 73.6
RIPV	218.1 ± 91.2
**Left atrial appendage closure**	
**LAA shape, n (%)**	
Cauliflower	19 (25.0)
Cactus	21 (27.6)
Chicken wing	21 (27.6)
Windsock	15 (19.7)
LAA opening diameter, mm	21.5 ± 3.4
LAA depth, mm	25.5 ± 4.5
Successful implantation, n (%)	75 (98.7)
**Devices, n (%)**	
Lefort	38 (50.7)
Lacbes	17 (22.7)
WATCHMAN	20 (26.7)
**Residual flow, n (%)**	
≤3 mm	15 (19.7)
>3 mm	0
**Complications**	
Vagal reflex, n (%)	5 (6.6)
Phrenic nerve palsy, n (%)	0
Cardiac tamponade, n (%)	0

*LAA, left atrial appendage; LIPV, left inferior pulmonary vein; LSPV, left superior pulmonary vein; RIPV, right inferior pulmonary vein; RSPV, right superior pulmonary vein*.

### Follow-Up

**Figure 4:**
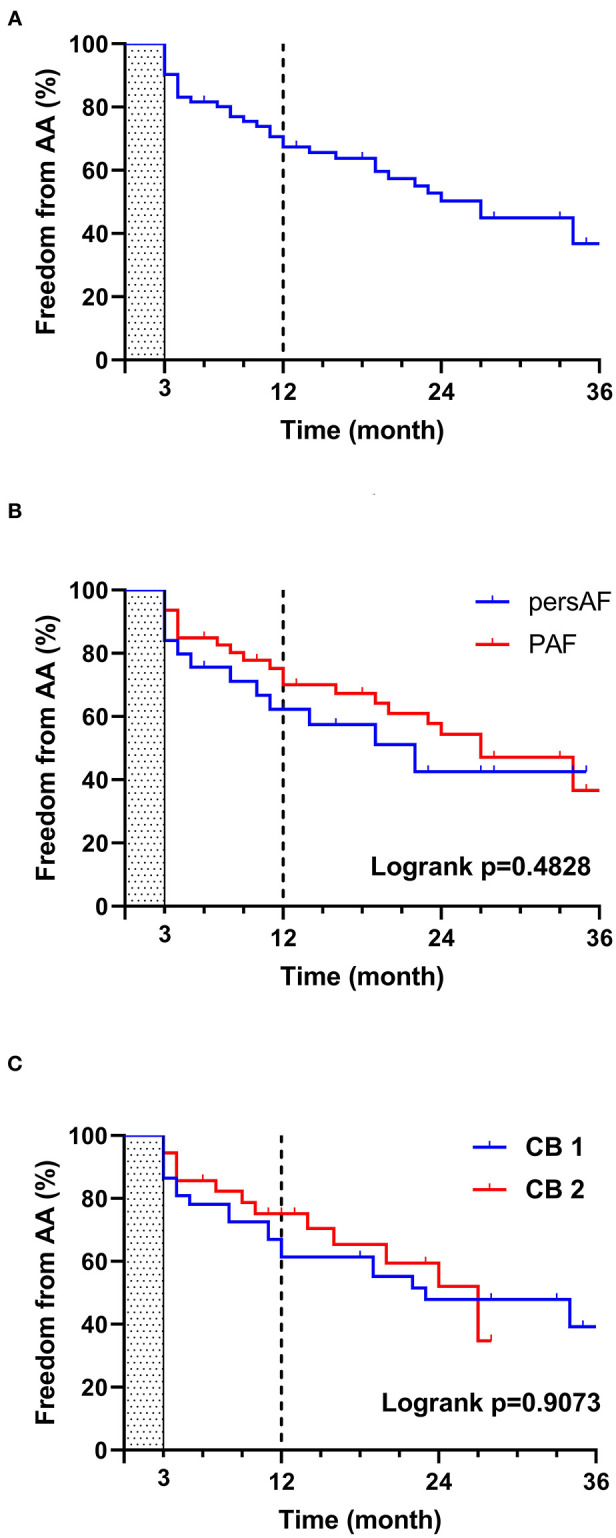
Freedom from atrial arrhythmia (AA) by survival analysis. The shaded area before the third month shows a blank period where recorded AA is not considered as a recurrence. The dashed black line shows the follow-up time of 12 months. **(A)** Overall freedom from AA, **(B)** freedom from AA grouped by AF type, **(C)** freedom from AA grouped by CB generation. AA, atrial arrhythmia; CB, cryoballoon (1 and 2 refer to the first and second generation, respectively); PAF, paroxysmal atrial fibrillation; persAF, persistent atrial fibrillation.

Through 23.7 ± 11.0 months of follow-up, contact was lost with three patients (4.0%). Among the patients who reached primary endpoints, 35 had AA recurrence, with a 1-year freedom from AA rate of 67.4%. No differences were found, however, in the subgroups divided by AF type and CB generation (as depicted in Figure 4); one had a lethal intracranial hemorrhage on SAPT.

Regarding secondary endpoint assessments, mortality was low and resulted from one case of acute myocardial infarction and one case of lethal hemorrhagic stroke, as mentioned above. TTE revealed mild to moderate pericardial effusion in three patients, two of whom recovered from pericardiocentesis. No other complications like systemic embolism or atrioesophageal fistula occurred during follow-up.

In addition, TEE was completed in 72 patients, after which one patient who had an implanted Lefort device saw a significant residual (10 mm) in the third month, though this dwindled to 6 mm in the 12th month. Thrombosis (13 × 4 mm) was detected on the device surface of one patient who had a Lacbes device implanted in the third month; significant resolution was lacking by the 12th month. Both of them were prescribed lifelong OACs. In addition, six patients had minimal residual flow. No displacement of devices was observed. As observed during follow-up, no severe adverse events occurred to these six patients, with the exception of one who was rehospitalized after complaining of chest distress 2 years after the procedure and thus diagnosed with coronary heart disease *via* coronary angiography. This patient was discharged with SAPT, and no adverse events occurred. Detailed outcomes are described in Table 3.

**Table 3:** Follow-up outcomes.

**Outcomes**	**Combined procedure (*N* = 73)**
**Primary endpoints**	
Atrial arrhythmia recurrence, n (%)	35 (48.0)
Stroke, n (%)	1 (1.4)
**Secondary endpoints**	
Death, n (%)	2 (2.7)
Rehospitalization due to cardiovascular causes, n (%)	28 (37.4)
Major hemorrhage, n (%)	1 (1.4)
Systemic embolism, n (%)	0
Myocardial infarction, n (%)	1 (1.4)
Redo-ablation, n (%)	4 (5.5)
Pericardial effusion, n (%)	3 (4.1)
Atrioesophageal fistula, n (%)	0
Oral anti-coagulants withdrawal, n (%)	2 (2.7)
**TEE examination**	
TEE completed, n (%)	72 (94.7)
Displacement, n (%)	0
Residual flow, n (%)	
≤3 mm	6 (8.2)
>3 mm	1 (1.4)
Device-related thrombosis, n (%)	1 (1.4)

*TEE, transesophageal echocardiography*.

**Figure 5:**
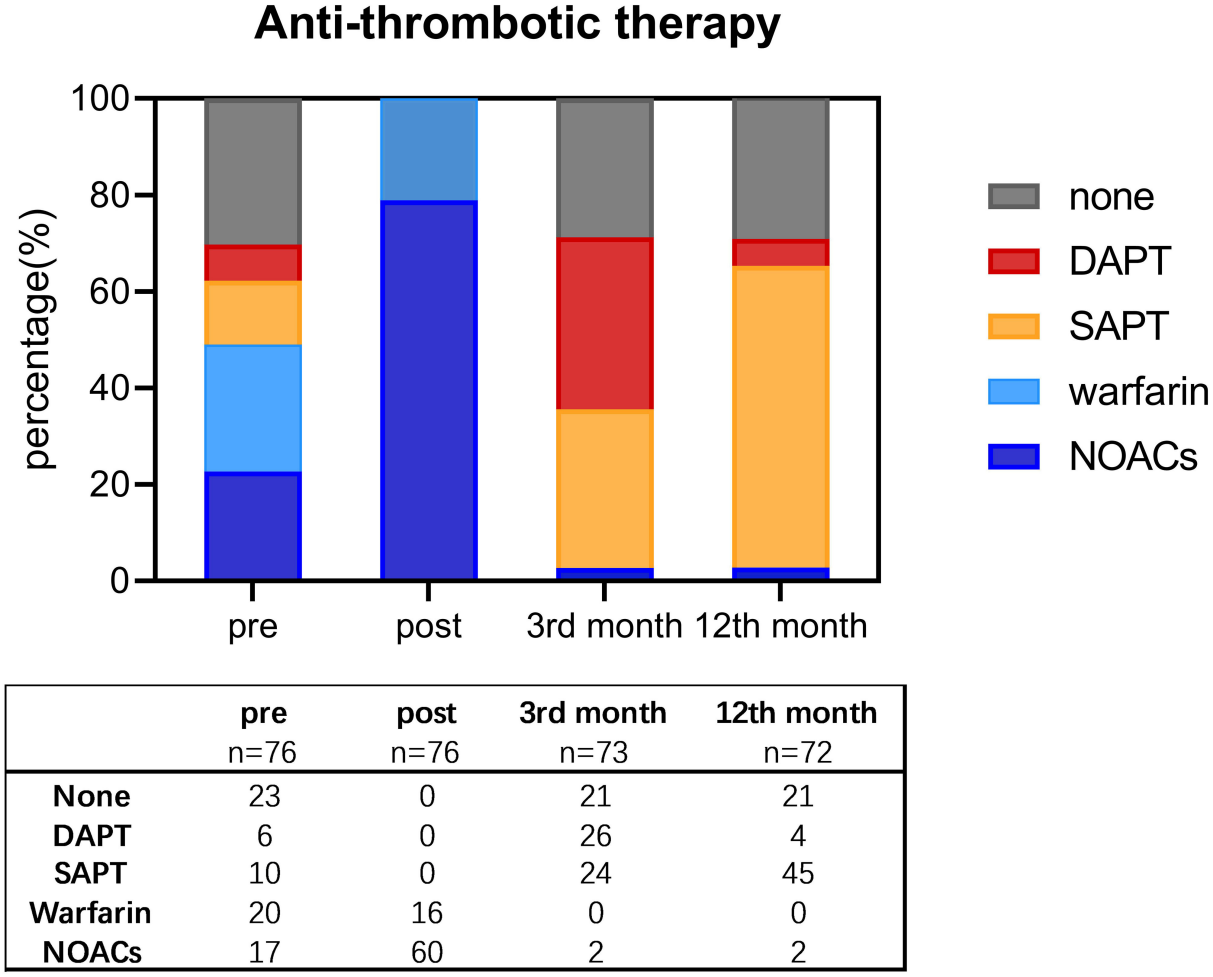
Shift of antithrombotic therapy before procedure, post-procedure, and by the third and 12th month since the procedure. DAPT, double antiplatelet therapy; NOACs, new oral anticoagulants; SAPT, single antiplatelet therapy.

Overall antithrombotic therapy showed a falling pattern, as depicted in Figure 5. Withdrawal of OACs was suggested in 70 patients after the confirmation of complete sealing by TEE in the 3rd month. Two patients discontinued OACs due to the device complications above, and one discontinued OACs due to a failed LAAC procedure. By the 12th month, only four patients were on DAPT, while other patients were on either SAPT or no antithrombotic therapy.

## Discussion

Our study presents the first long-term clinical outcomes in non-valvular, drug-refractory AF Chinese patients, the largest registered combined CBA procedure cohort worldwide. Our primary findings show that the combined CBA procedure in indicated patients can achieve (1) acceptable AF rhythm control in both PAF and persAF, (2) effective stroke prophylaxis with a high OAC withdrawal rate, and (3) considerable safety with a low mortality and complication rate.

Despite an AF prevalence of 10 million, AF in China is poorly managed, and anticoagulation in the country is far from sufficient. Indeed, one study reported only 11% of Chinese AF patients were on OACs (17), albeit the high incidence of stroke (18). In this study, only 48.7% of AF patients were on OAC treatments before admission, and they had a high previous stroke rate of 44.7%. Among the various reasons, non-compliance accounted for these insufficiencies to a great extent. Fortunately, since the advent of this combined procedure, short-term studies on Chinese populations have shown that AF can be managed with a high OAC withdrawal rate (19). Evaluation of the efficacy and safety of the combined procedure, however, still requires longer clinical observation.

### Efficacy

The combined procedure has been proven to have no influence on PVI or LAAC manipulation and outcomes (12). As shown in this study, the overall success rate of instant PVI with satisfactory LAAC is 97.2%. This parallels current studies on combined procedures (10–13) and a study specifically on the combined CBA procedure (14).

Regarding ablation, the reported recurrence rate of the combined procedure was comparable to that of studies using only the ablation procedure, where the first study on a combined procedure by Swaans et al. (10) presented a recurrence rate of 30% through 12 months of follow-up; the first study of the combined CBA procedure by Fassini et al. (14) had a reported a recurrence rate of 29% through 24 ± 12 months of follow-up. In the present study, an instant PVI was achieved in 75 patients, with the exception of one failure to isolate RIPV due to a mismatched balloon size and RIPV variation despite twice freezing the common trunk. This success rate is comparable with our solitary CBA results (16) and the combined CBA procedure outcomes by Fassini et al. (14). After over 2 years of follow-up, the freedom from AA rates by the 12th month (67.4%) and last follow-up (52.0%) are relatively low compared with those of current studies (10–14). According to previous studies, a large average LA diameter (20) and a higher proportion of persAF (21) contribute to a higher incidence of AF recurrence. In the present study, the average LA diameter was 44.9 ± 0.55 mm, and the proportion of persAF was 32.9%, which could account for the high recurrence rate during follow-ups. In addition, we uniformly performed PVI without touch-up ablation, which other studies have reported to be beneficial (22). Larger-scale prospective studies are warranted to provide guidance for ablation using CB during combined procedures.

Considering LAAC, previous studies have reported an instant complete occlusion rate of 100% and complete occlusion rates of 91–93% (10, 14). Similarly, we observed an instant, complete occlusion rate of 98.7% as well as a 3-month complete occlusion rate of 92.1%. Only one patient experienced WATCHMAN device deployment failure due to an oversized LAA. A WASP study showed that the average LAA ostium diameter was significantly larger in Asian populations, thus requiring larger WATCHMAN devices (23). Therefore, based on our experience, we recommend the thorough pre-procedural evaluation of LAA morphology as well as larger LAAC devices to accommodate LAA in Asian populations.

### Safety

Complications during the combined procedure were less than expected according to published results (10–14). The most frequent complications were mild, such as pericardial effusion and minimal residual flow (10, 14). Only the study by Swaans et al. (10) in 2012 reported three cases (10%) of major hemorrhaging through a 12-month follow-up. Similarly, our study showed a mortality of 2.7%, which resulted from one case of acute myocardial infarction on dabigatran in the third month and one case of lethal hemorrhagic stroke on aspirin in the 12th month. Both patients had successful combined procedures without complications. In addition, the major hemorrhage rate was 1.4%, which resulted from the hemorrhagic stroke mentioned above. This is in line with other published results on combined procedures, where major hemorrhaging occurred in 0–10% of the studied population (10, 14, 19). Our evidence shows that the combined CBA procedure can be safely performed in the Chinese population.

In addition, the TEE follow-up showed no displacement of devices, while six patients (8.2%) had minimal residual flow. Minimal residual flow—defined as ≤5 mm in the PREVAIL trial—was proven to have no correlation to device displacement or related thrombosis (8). In a recent meta-analysis by Han et al. (24), the occurrence of residual flow was associated with higher hemorrhagic risk. Although no similar correlation was found in our study, the definition and significance of residual flow demand further research.

Of note, one patient with a congenital atrial septal defect (7 mm) had an LAA opening of 30 mm and was implanted with a Lefort device of maximum size (33 mm). Although the procedure was completed with an acceptable compression rate of 81% and a minimal residual flow of 3 mm, this flow developed into a significant 10-mm residual flow in the third month and a 6-mm residual flow in the 12th month. We hypothesize that a mismatch between LAA and the device contributed to this occurrence. A large residual flow could possibly harbor thrombi and should be managed with either discontinued OACs or another LAAC device for stroke prophylaxis (25). In this study, since we prescribed lifelong rivaroxaban to this patient, no occurrence of embolization or hemorrhage was observed during follow-up. Based on our experience, we believe that a larger LAAC device is necessary in the Chinese population. Likewise, in the presence of significant residual flow, the discontinuation of OACs can be an effective remedy.

Another patient had a Lacbes device successfully implanted but, due to an interrupted OAC treatment, had an obvious DRT (15 × 8 mm^2^ by TEE) in the third month. Although having continued OAC, this thrombosis had remained at a similar size (13 × 4 mm^2^) by the 12th month. DRT was observed with an incidence of 4.2% in the PROTECT AF study with an annual stroke rate of 0.3% (26). Nevertheless, this was a rare occurrence in the published results for combined procedures. The current study shows that DRT is related to stroke and should be managed with OACs (25). Although thrombosis was still present, since we continued OAC, neither embolic events nor major hemorrhaging events occurred during follow-up. Based on our experience, we believe DRT should be managed with continuous OACs to prevent thromboembolic events.

The OAC withdrawal rate (97.2%) was high, as is reported in most combined procedure studies (10–14). Although we recommended 3 months of DAPT and lifelong SAPT following TEE examination, patients had a low adherence to antithrombotic therapy; 21 patients refused to take any antiplatelet agent due to either adverse drug effects or minor bleeding events, and only 26 patients underwent DAPT for 3 months. As of now, there is still a lack of consensus on an antithrombotic strategy following combined procedures. An OAC therapy adapted by current studies-−2–3 months following 6 months of DAPT and then lifelong SAPT—shows satisfactory stroke prophylaxis with low hemorrhagic risk (10–14, 19). Based on our observation, we recommend 3 months of OAC therapy followed by 3 months of DAPT and lifelong SAPT if satisfactory sealing *via* TEE occurs during the third month. Due to the limited follow-up time and the small scale of these studies, however, the occurrence of hemorrhagic or embolic events was too low to detect a significant difference between groups receiving standardized treatment and those exhibiting low adherence. Longer follow-ups and larger trials are needed.

## Limitations

First, although we observed a relatively low rate of complications and a high rate of AF control—showing how the selected population can benefit from a combined procedure—further well-designed, large-scale research is needed on the indicated populations that benefit from such a procedure. Second, we cannot extrapolate these conclusions for a combined procedure utilizing RF or other LAAC devices. Third, due to the limited scale of the indicated population, the sample size is too small to provide strong evidence for the benefits of the combined procedure or to detect a significant difference among different LAAC devices. Larger studies or multicenter studies are needed to support this conclusion. More importantly, these outcomes could have been overestimated, as only 72 patients had a TEE record. As TEE is intolerable for most patients during follow-up, a more moderate evaluation approach is warranted.

## Conclusions

Combining CBA with LAAC in a single procedure is feasible with acceptable long-term safety and efficacy. Further large-scale, prospective, controlled trials are warranted to optimize this combined procedure.

## Data Availability Statement

The raw data supporting the conclusions of this article will be made available by the authors, without undue reservation.

## Ethics Statement

The studies involving human participants were reviewed and approved by Ethics Committee of Shanghai Tenth People's Hospital. The patients/participants provided their written informed consent to participate in this study. Written informed consent was obtained from the individual(s) for the publication of any potentially identifiable images or data included in this article.

## Author Contributions

ZR and JiZ contributed to the interpretation of data for the work. ZR, JiZ, and SW contributed to drafting the work. PJ, XL, JuZ, HY, YZ, and WM contributed to the acquisition of data. RG, HL, and SL contributed to the analysis and revision of the work. YX and DZ contributed to the conception of the work. All authors contributed to the article and approved the submitted version.

## Conflict of Interest

The authors declare that the research was conducted in the absence of any commercial or financial relationships that could be construed as a potential conflict of interest.
